# Time domain self-bending photonic hook beam based on freezing water droplet

**DOI:** 10.1038/s41598-023-34946-7

**Published:** 2023-05-12

**Authors:** Oleg V. Minin, Igor V. Minin, Yinghui Cao

**Affiliations:** 1 Nondestructive Testing School, Tomsk Polytechnic University, 36 Lenin Avenue, Tomsk, Russia 634050; 2 College of Computer Science and Technology, Jilin University, 2699 Qianjin Street, Changchun, 130012 China

**Keywords:** Materials science, Optics and photonics, Physics

## Abstract

Tunable optical devices are of great interest as they offer adjustability to their functions. Temporal optics is a fast-evolving field, which may be useful both for revolutionizing basic research of time-dependent phenomena and for developing full optical devices. With increasing focus on ecological compatibility, bio-friendly alternatives are a key subject matter. Water in its various forms can open up new physical phenomena and unique applications in photonics and modern electronics. Water droplets freezing on cold surfaces are ubiquitous in nature. We propose and demonstrate the effectual generation of time domain self-bending photonic hook (time–PH) beams by using mesoscale freezing water droplet. The PH light bends near the shadow surface of the droplet into large curvature and angles superior to a conventional Airy beam. The key properties of the time–PH (length, curvature, beam waist) can be modified flexibly by changing the positions and curvature of the water–ice interface inside the droplet. Due to the modifying internal structure of freezing water droplets in real time, we showcase the dynamical curvature and trajectory control of the time–PH beams. Compared with the traditional methods, our phase-change- based materials (water and ice) of the mesoscale droplet have advantages of easy fabrication, natural materials, compact structure and low cost. Such PHs may have applications in many fields, including temporal optics and optical switching, microscopy, sensors, materials processing, nonlinear optics, biomedicine, and so on.

## Introduction

A photonic hook (PH)^1^ is a new localized high-intensity curved light, representing the smallest subwavelength radius of curvature of any electromagnetic beam, focused by a dielectric mesoscale (i.e. diameter in order of wavelength) particle. Now it is the only example of artificial light bending apart from the Airy-family beam^2^ since it was invented in 2015^3^. The interference (superposition) of the transmitted, diffracted and scattered waves in the shadow part of the particle with broken symmetry have intriguing properties demonstrating the effect of curving in space. The inflexion point where the curved localized beam changes its propagating direction is an important characteristic of the PH which is not possessed by Airy-like family beams. PH has unique features—the radius of curvature is a subwavelength and this is the smallest ever reported curvature of electromagnetic beams^1^. Several types of asymmetry are known that lead to the formation of a photonic hook^1^, in contrast to the Airy-family beams which are commonly created using dynamic diffractive devices or static phase plates^4^. There they are the asymmetry of the particle shape, the asymmetry of the optical properties of the material of the particle with its symmetrical shape^1^, the asymmetry of the radiation incident on the particle^1,5^ and the asymmetry of the medium surrounding the particle in its shadow part^6^.

Typically, the material of the particle is a dielectric (including liquid) or an artificial material^1^. At the same time, the important material that plays a key role in wildlife is water. According to Leonardo Da Vinci “Water is the driving force of all nature”. Nature has developed objects, materials and processes in a wide-scale size up to the meso- and nano- scale. The mimic of nature allows to develop processes, devices and materials which provide desirable properties. These will lead to green technology and science. Sometimes nature provides us with optical focusing structures, for free. Water droplets are ubiquitous in living nature and play a crucial role in many biological, physical and industrial processes. For example, water droplets were the first lenses found by people about six thousand years ago, it was known that small water droplets burn leaves through^7,8^.

The optical effects in water droplet were examined in^9–15^. Optical backscattering from water droplets with diameter of 6–90 um based on Van de Hulst's theory was studied in^16^, a water droplet resonator was investigated in^17^. In^18^ it was shown that a spherical 30–70 um water droplets can focus a femtosecond pulse (a few μJ per pulse at 810 nm) of light near its shadow surface. At this inner focus, water molecules ionize and heat up to the temperature about *T* ~ 5000 … 7000 K, create a localized area of plasma that emits a beam of white light 35 times as intense back toward the illumination source. Similar effects can be expected in the superresonance mode^15^.

Freezing or melting water drop as a phase change material (PCM)^19,20^ also has a long history. The physical effects in freezing water droplet is a problem of fundamental importance which was the subject of numerous studies, and references thereto have been found since the time of Aristotle^21–25^. The water–ice phase transitions were also widely studied^26–30^.

The temperature-dependent optical properties of water enable to dynamically tune and reconfigure water-droplet- based devices. In this work, we offer and demonstrate the concept of self-bending photonic hook beam in the time domain, providing a new direction in temporal optics. We focus on time–PH that is based on a cooled mesoscale water drop (or evaporation of ice droplet). For the first time we study in detail the possibility and features of the dynamical formation of a photonic hook. The idea of such a consideration was offered in^31^. The phase state of water drop changes from liquid to solid in the process of freezing. These materials have different optical properties^32^ giving rise to the asymmetry of optical properties of the particle material, and formation of a photonic hook and photonic jet (PJ). Accordingly, freezing mesoscale droplets will show the potential to exploit the dynamic properties of the PH in optical all-dielectric devices.

## Model

**Figure 1:**
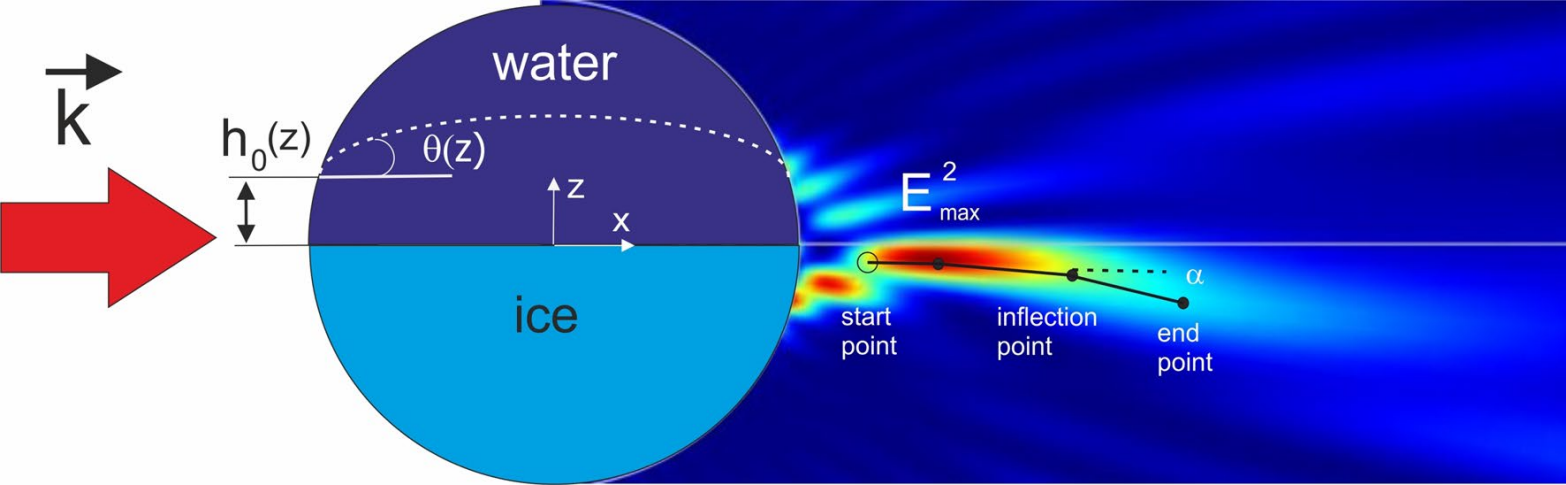
A schematic diagram of the photonic hook formation by freezing water droplet. Black points show the positions of the inflection points along the PH, h_0_—position of the water–ice interface.

The photonic hook formation by cooled water mesoscale spherical particles immersed in air (n = 1) was simulated based on the finite elements method (FEM) by using the commercial software COMSOL Multiphysics. Spherical shape of the droplet corresponds to a small Bond number, which represents the relations of surface tension and liquid gravity. In simulation, 2D geometry^33^ and a non-uniform mesh were employed to reduce the computational time and cost. As the boundary condition, the Perfect Matched Layer (PML) was applied. The incident light with a linear polarization along the y-axis was assumed to be a plane wave that propagates along the x-axis. The indices of water and ice are 1.334 and 1.301, respectively, at the wavelength of λ = 589 nm. Inside and outside the water–ice drop, the mesh size is λ/15 and λ/8, respectively. A schematic diagram is shown in Fig. 1.

Droplet freezing dynamics is a multistage process. When a drop of water is placed on a cold surface (for example, refrigerator, airplanes, electric cables, wind turbines, and so on), the heat is transferred from that surface to the water drop. Freezing of the droplet on the cold substrate does not occur at once: the frozen water–ice phase boundary interface moves inward from the droplet–substrate interface toward the droplet free surface^34–38^. The water droplets freezing speed is independent of the thermal conductivities of the substrates and increases with the decrease of the substrates temperature^39^. To simplify the problem, in our scenario, following^40^, both the thermal conductivity of the environment, and the convective heat transfer are low. For the liquid at the water–ice interface isothermal condition is assumed^41^. It is also assumed that liquid and solid water (ice) are monolithic materials without any inclusions and inhomogeneities.

A simple model for the shape of the ice/water interface during the freezing process of water droplet was discussed in^42^. The propagation and the shape of the ice/water interface depend on the characteristics of heat transfer inside the drop. However the quantitative model that is able to predict the shape and interface of ice/water drops where the solid, liquid and vapor phases meet^43–45^, is not fully understood now and is beyond the scope of this paper. What’s more, in^42^ it was shown that the freezing along the surface occurs in the direction along the ice/water interface, and may be characterized by the contact angle θ = θ(R, ${\varvec{v}}$)—(Fig. 1), where $v={\rho }_{s/{\rho }_{l}}$ is the density ratio (${\rho }_{s}$ and ${\rho }_{l}$ are the solid and liquid densities, respectively). In this simple model we did not take into account the thin layer of liquid (so-called quasi-liquid layer) on the surface of ice near the triple point^45^. Although during the freezing process the interface may change its shape, we assume that the water–ice interface keeps the bending surface with fixed curvature radius. Note that a spherical water–ice interface front that meets the edges of the drop perpendicularly for freezing water drops on a copper substrate was experimentally and theoretically considered in the latest paper^46^. In addition in^47^ it was shown both theoretically and experimentally that the ice–water front of water droplets deposited on a surface at subzero temperatures becomes concave for big (centimeter scale) droplets. Also the shape of both the droplet and the ice at the bottom take a spherical shape which is possible by using superhydrophobic surfaces^33,48,49^. Nearly spherical shape of a frozen water droplet was observed experimentally in pure water evaporatively cooled in a vacuum^24^ and in the water droplet on silver nanocolumnar thin film^50^. But most of the drops freezing experimental research has been carried out with large drop sizes in millimeter scale.

It could be noted that from this point of view, such freezing droplet may be considered as Janus^51^ time-dependent mesoscale^52^ particle. There are two additional degrees of freedom which makes it possible to control the characteristics of a localized PH, namely, curvature and position of the water/ice interface inside the droplet during the freezing process.

**Figure 2:**
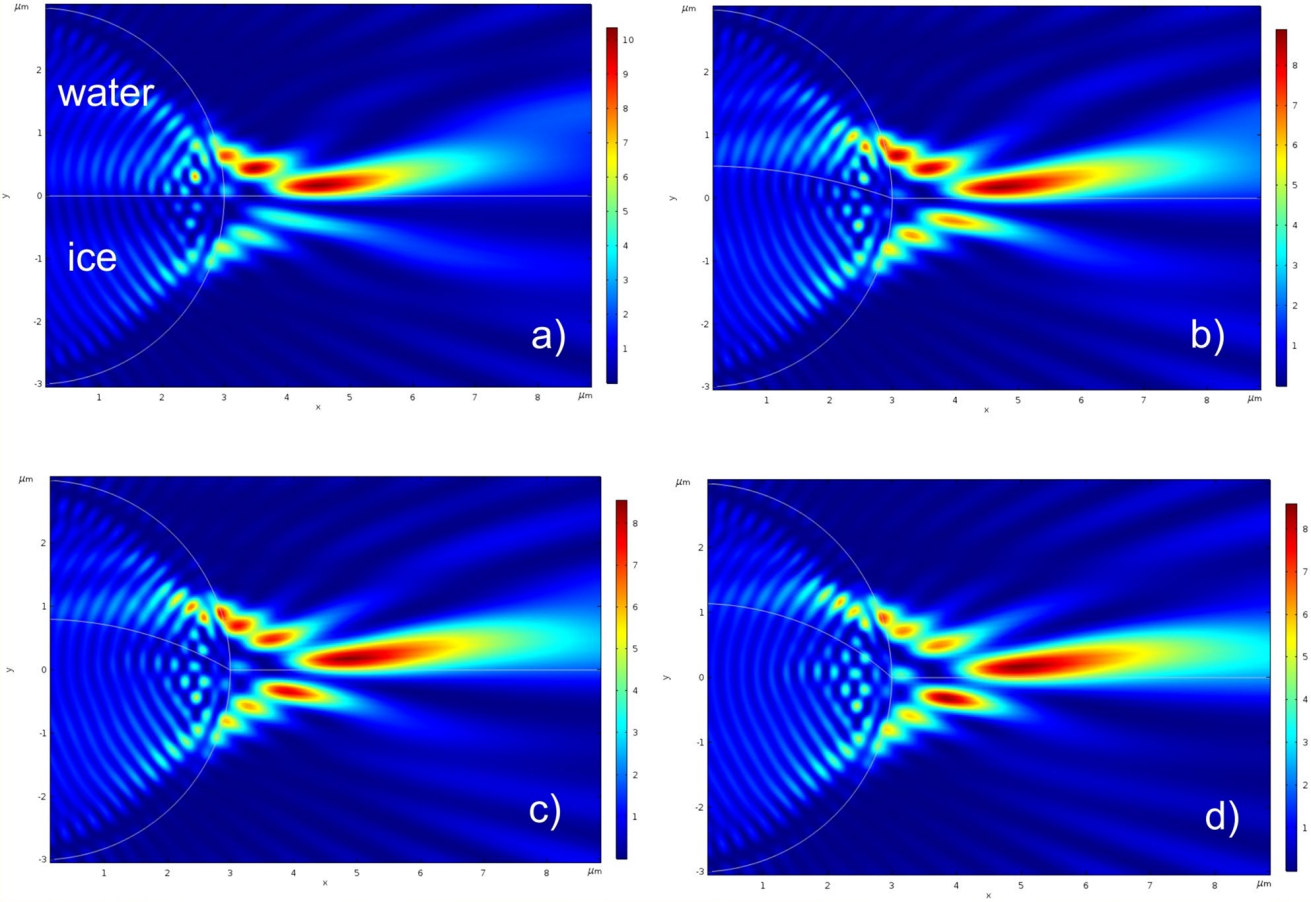
Normalized to illuminated wave light intensity distributions of the PHs formed by the frozen water droplet for different curvature of water–ice interface: (**a**) flat water–ice interface, (**b**) R_c_ = 1.5R, (**c**) R_c_ = 2R, (**d**) R_c_ = 3R. The Comsol software (v.5.3, https://www.comsol.com/) were used to create the images.

The evolution of the photonic hook shape for different curvatures of the water–ice interface R_c_ at a fixed position of h = 0 (see Fig. 1) is shown in Fig. 2 for a drop with a radius of 6 microns. Such water drops, for example, are present in clouds and fog^53^. The curvature of the photonic hook under the initial definition^1,54^ is approximately determined by the $\alpha$-factor. To characterize this factor, called the bending angle of PH, we introduced the position of the “inflection point”^55^ where the electric field intensity I_max_ = max(|E|^2^) along the PH had maximum, for the first time in 2018^1,56^. Usually it is the angle between the two lines linking the start point with the inflection point and inflection point with the end point of the PH, respectively (see Fig. 1). At the same time, depending on the specifics of the problem, there may be several inflection points along the propagation of the photonic hook. That is, several inflection points can be observed where the photonic flux changes its direction. In our case, we analyze three such points.

## Results and discussion

For evaluating positions of the inflection points along the PH and the bending angles $\alpha$ of the PH (see Fig. 1), we use the following simple and practical method^1^. First, the image of the field intensity distribution in the shadow part of the particle is obtained and a contour map is constructed at the level 1/e of the maximum intensity peak in the vicinity of the photonic hook. Then, the points of change in the direction of propagation of the photonic hook are determined, and the left and right PH’s arms are determined with an inflection point relative to the point with maximum intensity I_max_ along the PH. Next, the end and start points are selected as the extreme points of both the right and left arms, respectively, relative to the point with I_max_. This algorithm in more detail is described in^1^.

Despite the weak optical contrast between the refractive indices of water and ice (n_c_ = 1.334/1.301 = 1.025), even with a flat interface between these two media, a photonic hook is formed in the shadow part of the spherical drop (Fig. 2a). An increase in the curvature of the interface leads to an increase in the length of the photon hook (Fig. 2b–d). At the same time, it is clearly seen that the propagation path of the beam is deflected at the inflection point with I_max_ = max(|E|^2^), resulting in bending of PH.

**Figure 3:**
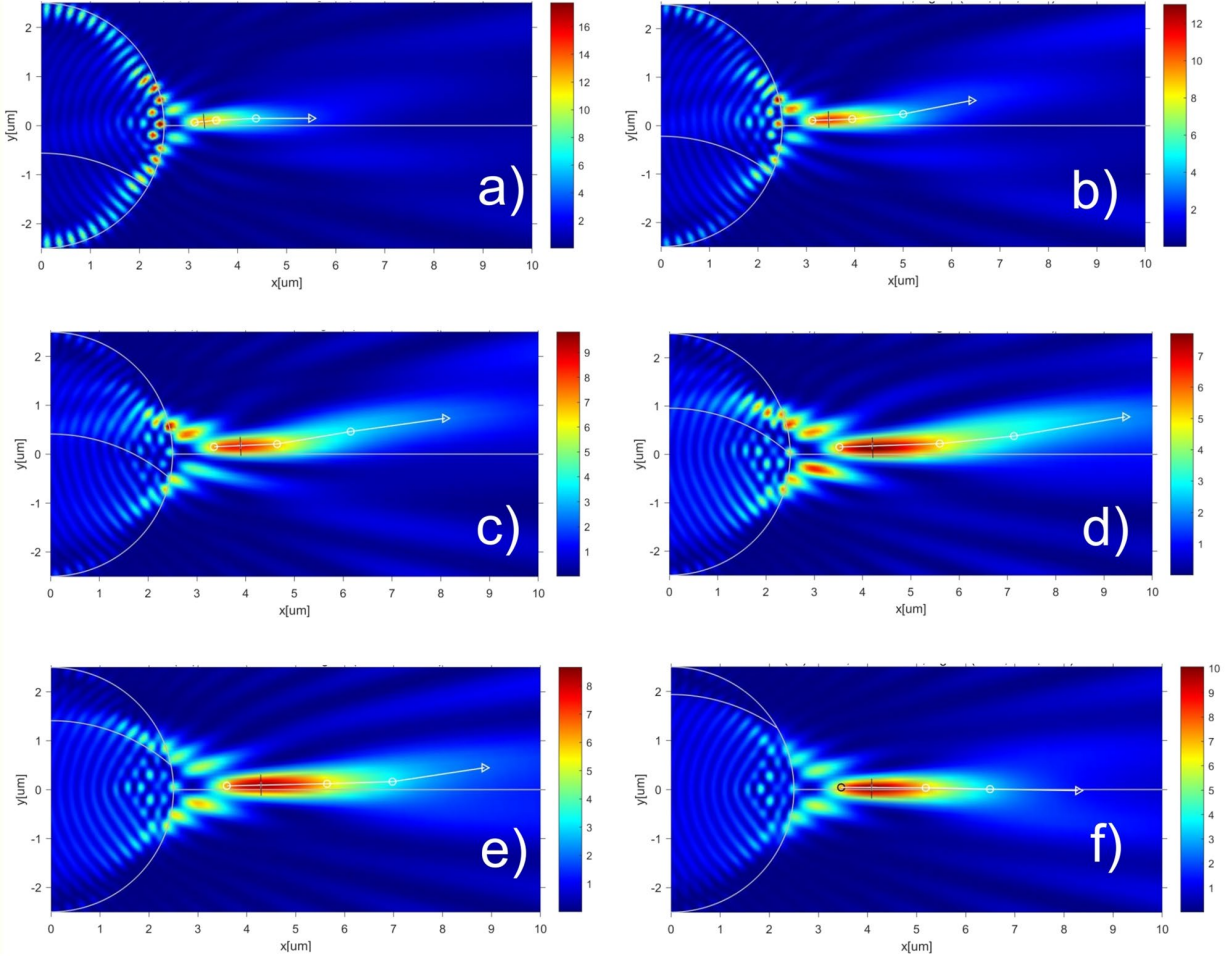
Formation of the PH with water–ice interface curvature radius R_c_ = 1.5R and with different position of water–ice interface: (**a**) h/R =  − 0.5; (**b**) h/R =  − 0.4; (**c**) h/R =  − 0.2; (**d**) h/R = 0; (**e**) h/R = 0.2; (**f**) h/R = 0.5. The short lines in black color represent the FWHM of the focused light beam. The Comsol software (v. 5.3, https://www.comsol.com/) were used to create the images.

Let us now consider the features of the formation of a photonic hook for drops of smaller diameter^57^ depending on the position and curvature of the water–ice interface. The simulation results for the drop with radius R = 2.5um and with the water–ice interface curvature radius R_c_ = 1.5R are presented in Fig. 3. The main key characteristics of the PHs are shown in Table 1.

**Table 1:** Key characteristics of the PHs for R_c_ = 1.5R.

h/R	Max(E^2^)	FWHM	$\alpha$ 1, deg	$\alpha$ 2, deg	$\alpha$ 3, deg
− 0.5	13.05	0.3	5.53	0	2.17
− 0.4	11.14	0.35	1.97	0.77	14.72
− 0.3	9.93	0.36	2.82	6.35	12.48
− 0.2	8.80	0.39	2.52	9.64	7.95
− 0.1	8.11	0.41	2.31	5.19	9.98
0	7.94	0.43	1.96	5.82	9.81
0.1	8.10	0.43	1.61	7.13	11.24
0.2	8.66	0.43	1.19	1.83	8.62
0.3	9.27	0.43	− 0.43	0	− 0.8
0.4	9.72	0.41	0.46	− 0.64	− 0.31
0.5	10.14	0.4	− 0.28	− 1.18	− 0.96

As follows from the simulation results, the water–ice interface moves upward from the lower boundary of the droplet, when the length of the photonic hook increases due to the increase in the proportion of ice with a lower refractive index in the droplet. In this case, the position of the interface between the two media is of great importance. So, when the extreme boundaries of the water–ice interface coincide with the drop diameter (Fig. 3d), the length of the photonic hook is maximum. As the interface boundary moves further towards the top of the drop, the length of the photonic hook begins to decrease, its curvature decreases and tends to the shape of a photonic jet (Fig. 3f). The dynamics of the time–PH formation in this case is shown in supplement video 1.

From Table 1 it follows that the bending angles $\alpha$=$\alpha$(h/R) are nonlinear functions that go from negative to positive and vice versa.

**Figure 4:**
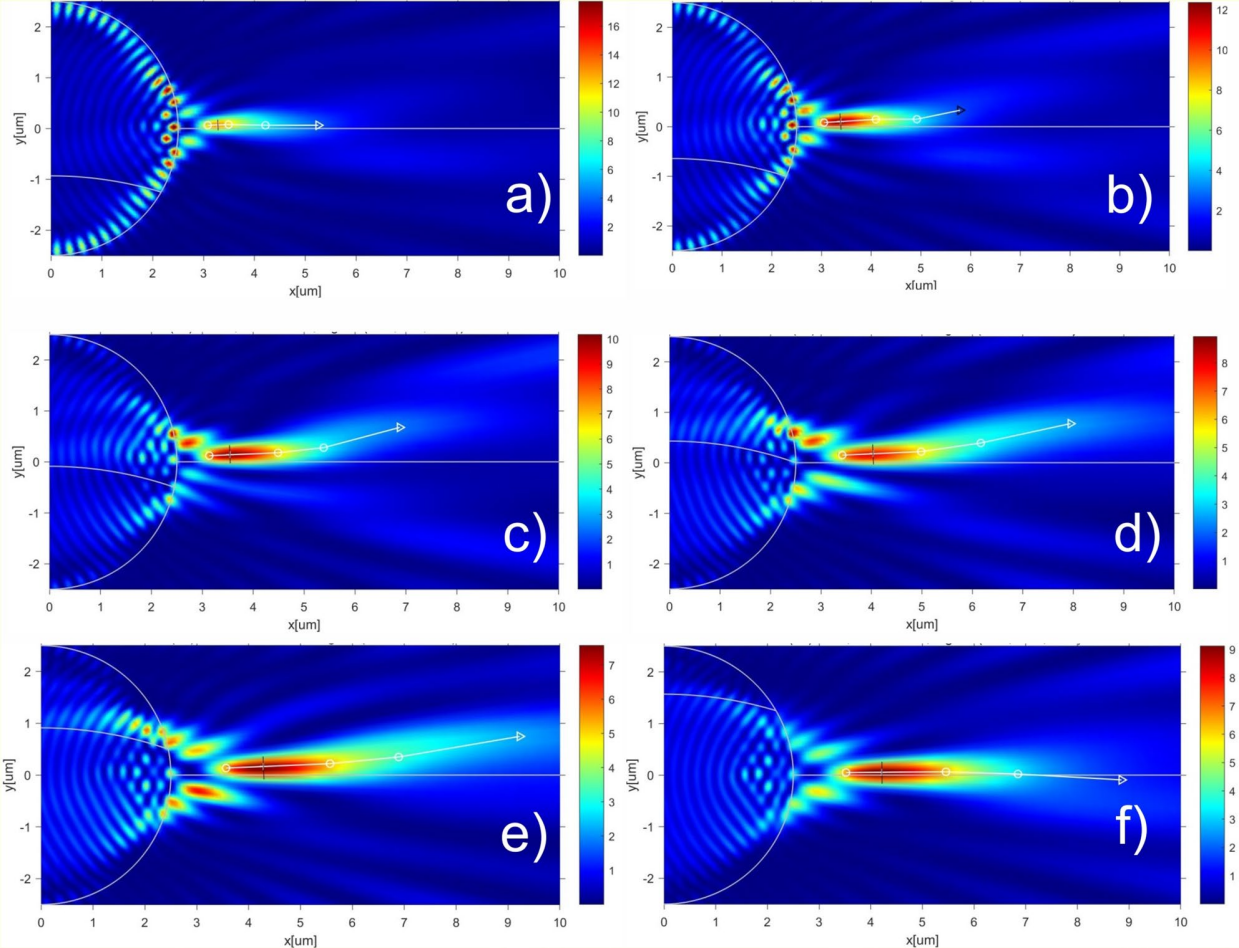
Formation of the PH with water–ice interface curvature radius R_c_ = 3R and with different position of water–ice interface: (**a**) h/R =  − 0.5; (**b**) h/R =  − 0.4; (**c**) h/R =  − 0.2; (**d**) h/R = 0; (**e**) h/R = 0.2; (**f**) h/R = 0.5. The short black color lines represent the FWHM of the focused light beam. The Comsol software (v.5.3, https://www.comsol.com/) were used to create the images.

Similar trends are observed for the increased curvature of the water–ice interface. The simulation results for the drop with radius R = 2.5um and with the doubled water–ice interface curvature radius R_c_ = 3R are presented in Fig. 4. The correspondent main key characteristics of the PHs are shown in Table 2. The dynamics of the time–PH formation for R_c_ = 3R is shown in supplement video 2.

**Table 2:** Key characteristics of the PHs for R_c_ = 3.0R.

h/R	Max(E^2^)	FWHM	$\alpha$ 1, deg	$\alpha$ 2, deg	$\alpha$ 3, deg
− 0.5	13.36	0.21	1.97	− 1.12	0
− 0.4	12.11	0.33	3.61	− 1.89	0
− 0.3	11.21	0.35	2.79	5.01	13.04
− 0.2	10.26	0.36	2.41	6.34	14.93
− 0.1	9.06	0.38	2.39	8.33	9.52
0	8.12	0.4	2.60	8.23	12.19
0.1	7.70	0.41	3.79	7.38	9.08
0.2	7.72	0.44	2.44	5.53	9.69
0.3	8.17	0.45	1.64	2.49	10.14
0.4	8.79	0.43	1.19	1.43	6.47
0.5	9.34	0.43	0.42	− 1.75	− 3.25

**Figure 5:**
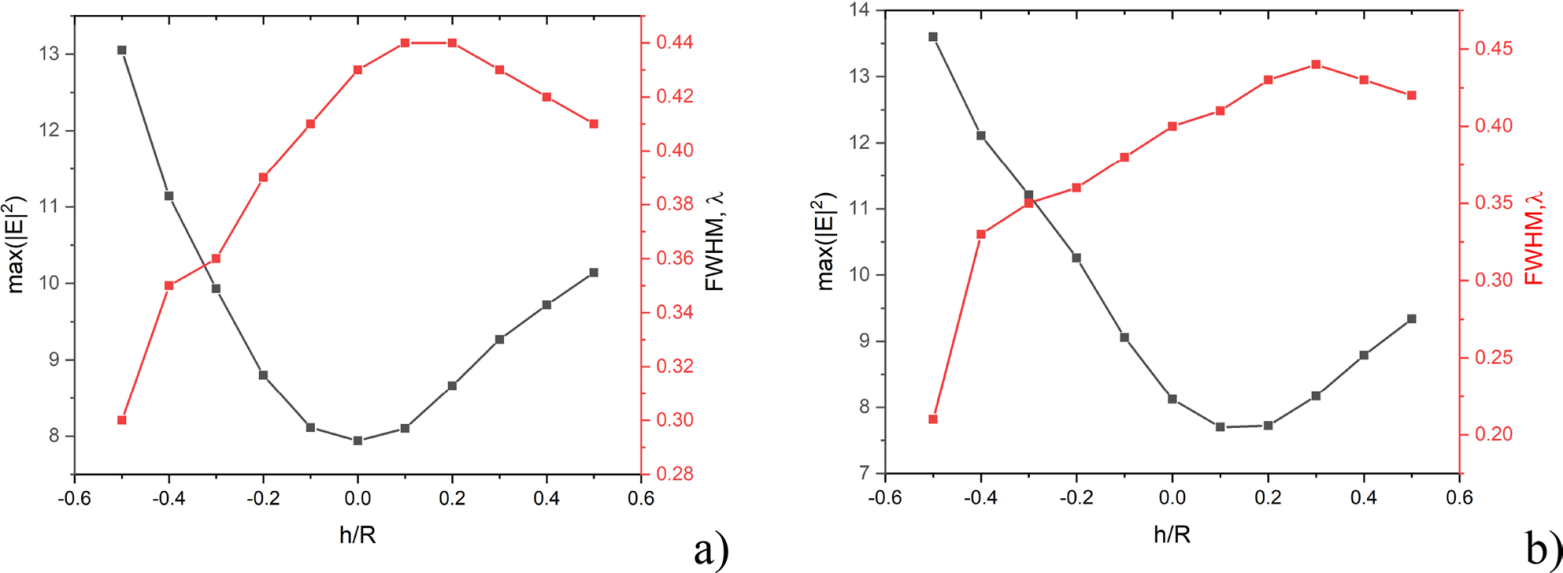
Maximal field intensity and FWHM of the PH vs h/R parameter for the water–ice interface curvature radius R_c_ = 1.5R (**a**) and R_c_ = 3R (**b**).

Analysis of the results presented in Figs. 3, 4 and in Tables 1, 2 makes it possible to plot the dependences of the maximum field intensity along the photonic hook and its beam waist size vs the h/R parameter. These data are presented in Fig. 5.

From Fig. 5 one can see that as the water–ice interface moves from the bottom of the drop to the top, the maximum field intensity along the photonic hook decreases and is minimal at h/R = 0 (Fig. 5a). When the interface boundary curvature doubles, the field intensity minimum shifts to the right, and the intensity drop is almost linear (Fig. 5b). The dependence of the width of the photonic hook (FWHM) at the point of maximum field intensity on the parameter h/R is also non-linear. But it is noteworthy that in the entire range of parameters, the minimum FWHM of the photonic hook is less than the simple diffraction limit, i.e. less than the half of the wavelength. It can also be seen that with an increase in R_c_, the minimum of the maximal field intensity of the photonic hook shifts to the large h/R values as the maximal value of the FWHM.

**Figure 6:**
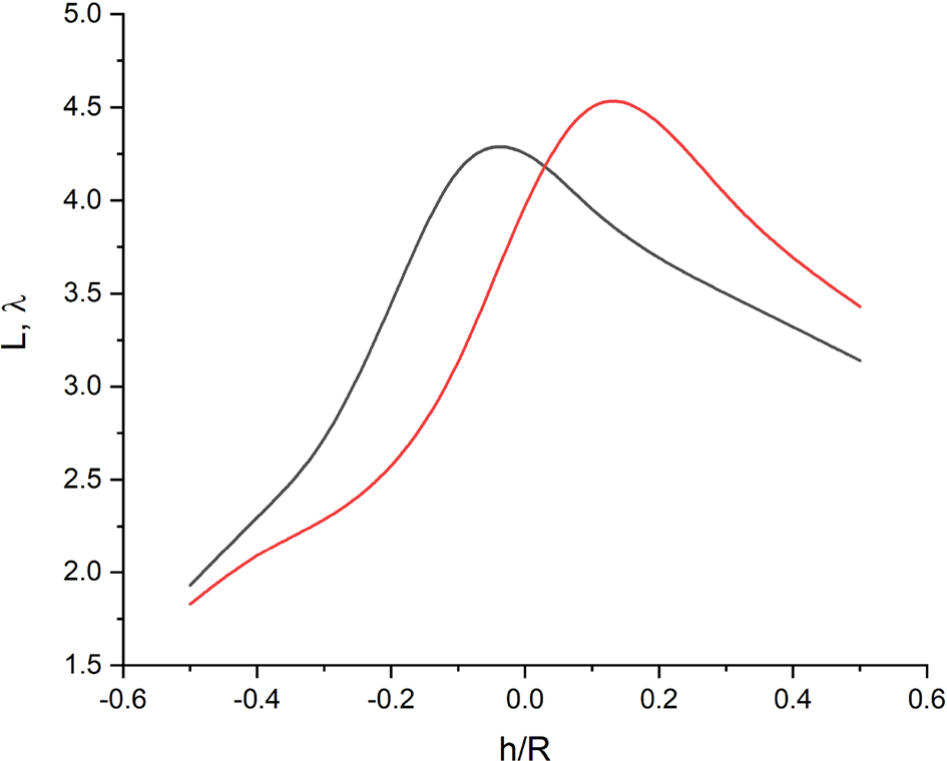
PH length vs h/R parameter for the water–ice interface curvature radius R_c_ = 1.5R (black) and R_c_ = 3.0R (red).

The length of the photonic hook (which is calculated by summing the line segment lengths between the start and the end points via the inflection point) has a pronounced maximum near the h/R = 0 (see Figs. 3d and 4d) value and is approximately 4.5λ both for R_c_ = 1.5R and R_c_ = 3.0R, which is shown below in Fig. 6.

It can also be seen that with an increase in R_c_, the maximum length of the photonic hook shifts to the right (to large h/R values) with a slight increase in the absolute value of its length. Note that it is possible to change or control the characteristics of a photonic hook based on a freezing water drop, for example, by increasing the ambient pressure, which will lead to an increase in the water–ice refractive index contrast^58^ or by using oblique illumination or placing the drop on an inclined surface^59^. Note that even if for some types of surface the shape of the water–ice interface inside the drop is flat rather than spherical, the formation of a photonic hook can be obtained by simply changing the angle of inclination of the incident radiation^1,60,61^. This discrepancy definitely calls for future investigation.

## Conclusions

The insights on the underlying mechanisms of water droplets dynamics during their freezing process will pave the way for a plethora of novel applications in a wide range of fields, including temporal photonics^62,63^, sensors, temperature and ice thermal storage control and biomedical engineering. Moreover, the understanding of freezing water droplet effects is a problem of general utility and fundamental importance which facilitates new applications of light localization non-resonance effects (such as photonic jets and hooks) in the field of optical devices and systems.

We demonstrate the concept of temporal photonic hook (time–PH) that is based on a cooled mesoscale water drop (or evaporation of ice droplet^64^) and will open a new avenue in temporal optics. It was shown that the freezing mesoscale water droplet makes it possible to focus the optical beam at the shadow part of the droplet into the photonic hook with different curvature despite low optical contrast between water and ice. The relationship of the refractive index contrast of liquid and solid water and the positions and curvature of the water–ice interface is found to form the PH with waist below the diffraction limit and bending angle on the shadow side of the freezing droplet.

By controlling parameters such as the size of the drop, the type of surface on which it is located^28^, including inclined surface, the ice fraction, different scenarios of freezing of a spherical water droplet^65,66^ etc., droplet freezing parameters can be controlled. In this case, the freezing time can be considered, for example, as one of the parameters of dynamic control over the characteristics of the photonic hook. It could be noted that taking into account the results of the previous study^32^, the current research may be extended into 3D case.

We believe that freezing- water- droplet- based elements are at the beginning of the research boom in photonics. In the long term this area will be determined by the goals for human-friendly environment proclaimed by the UN. It is noted annually by the UN^67^ on the ”World Water Day”, March 22.

Our results showcase the potential of the freezing water-droplet-based devices, which are to be bio-friendly, cheap, simple and dynamically tunable alternatives for many new optical applications^68–71^. This paves the way for freezing- water- droplet-based time–PH beam shaping, adding a new avenue in the road map of near-field structured light in mesotronics. We hope that our work will allow the use of freezing water droplets in optomechanics, optical sensors, and nanoparticle trapping and manipulation by means of both time-PJs and time-PHs in devices made from strictly natural liquid.

## Supplementary Information

Supplementary Legends.

Supplementary Video 1.

Supplementary Video 2.

## Data Availability

All data are available in the main text or upon a reasonable request from Corresponding Author.

## References

[1] O. V. Minin, I. V. Minin. *The Photonic Hook: From Optics to Acoustics and Plasmonics*. Springer Cham (2021).

[2] Dholakia K, Bruce GD (2019). Optical hooks. Nat. Photon..

[3] Minin IV, Minin OV (2016). Diffractive Optics and Nanophotonics: Resolution below the Diffraction Limit.

[4] Efremidis NK, Chen Z, Segev M, Christodoulides DN (2019). Airy beams and accelerating waves: An overview of recent advances. Optica.

[5] Minin IV, Minin OV, Liu Y-Y, Tuchin VV, Liu C-Y (2021). Concept of photonic hook scalpel generated by shaped fiber tip with asymmetric radiation. J. Biophoton..

[6] Minin OV, Minin IV (2021). Optical phenomena in mesoscale dielectric particles. Photonics.

[7] Beech M (2012). The Physics of Invisibility: A Story of Light and Deception.

[8] Egri Á, Horváth Á, Kriska G, Horváth G (2010). Optics of sunlit water drops on leaves: Conditions under which sunburn is possible. New Phytol..

[9] Gumprecht RO, Sliepcevich CM (1953). Scattering of light by large spherical particles. J. Phys. Chem..

[10] Nye, J. F. “Optical caustics in the near field from liquid drops. *Proc. R. Soc. Lond. Ser. A Math. Phys. Sci.* **361**(1704), 21–41 (1978).

[11] Lock J, Woodruff J (1989). Non-Debye enhancements in the Mie scattering of light from a single water droplet. Appl. Opt..

[12] Kolwas M (2010). Scattering of light on droplets and spherical objects: 100 years of Mie Scattering. Comp. Meth. Sci. Tech..

[13] Laven P (2011). Time domain analysis of scattering by a water droplet. Appl. Opt..

[14] Jacobsen, R. E., Arslanagić, S., & Lavrinenko, A. V. Mie resonances in water spheres for microwave metamaterials and antennas. *URSI Radio Sci. Lett.*, V. 1, (2019).

[15] Minin IV, Minin OV, Song Z (2022). High-order fano resonance in a mesoscale dielectric sphere with a low refractive index. JETP Lett..

[16] Saunders, M. J. Near-field backscattering measurements from a microscopic water droplet. J. Opt. Soc. Am. **60**(10), 1359 (1970).

[17] Qiao Z, Gong X, Guan P, Yuan Z, Feng S, Zhang Y, Kim M, Chang GE, Chen Y-C (2021). Lasing action in microdroplets modulated by interfacial molecular forces. Adv. Photon..

[18] Favre C, Boutou V, Hill SC, Zimmer W, Krenz M, Lambrecht H, Yu J, Chang RK, Woeste L, Wolf J-P (2002). White-light nanosource with directional emission. PRL.

[19] Zhang W, Mazzarello R, Wuttig M, Ma M (2019). Designing crystallization in phase-change materials for universal memory and neuro-inspired computing. Nat. Rev. Mater..

[20] Lone MI, Jilte R (2021). A review on phase change materials for different applications. Mater. Today Proc..

[21] Aristotle, Meteorology 350 B.C.E: http://classics.mit.edu/Aristotle/meteorology.1.i.html.

[22] Cheng RJ (1970). Water drop freezing: Ejection of microdroplets. Science.

[23] Zhang X, Huang Y, Ma Z, Zhou Y, Zhou J, Zheng W, Jiang Q, Sun CQ (2014). Hydrogen-bond memory and water-skin supersolidity resolving the Mpemba paradox. Phys. Chem. Chem. Phys..

[24] Wildeman S, Sterl S, Sun C, Lohse D (2017). Fast dynamics of water droplets freezing from the outside in. Phys. Rev. Lett..

[25] Ando K, Arakawa M, Terasaki A (2018). Freezing of micrometer-sized liquid droplets of pure water evaporatively cooled in a vacuum. Phys. Chem. Chem. Phys..

[26] Urray WAM, List R (1972). Freezing of water drops. J. Glaciol..

[27] Karlsson L, Ljung A-L, Lundstreom TS (2018). Modelling the dynamics of the flow within freezing water droplets. Heat Mass Transf..

[28] Karlsson L, Lycksam H, Ljung A-L, Gren P, Lundstrem TS (2019). Experimental study of the internal flow in freezing water droplets on a cold surface. Exp. Fluids.

[29] Hakimian A, Mohebinia M, Nazari M, Davoodabadi A, Nazifi S, Huang Z, Bao J, Ghasemi H (2021). Freezing of few nanometers water droplets. Nat. Commun..

[30] Xu, M., Gao, Y., Fang, F., Akhtar, S., Chaedir, B. A., & Sasmito, A. P. Experimental and unified mathematical frameworks of water-ice phase change for cold thermal energy storage. *Int. J. Heat Mass Transf*. **187**, 122536 (2022).

[31] Minin, I. V. & Minin, O. V. Dielectric particle-based strategy to design a new self-bending subwavelength structured light beams. *IOP Conf. Ser. Mater. Sci. Eng.* **1019,** 012093 (2021).

[32] Thormählen I, Straub J, Grigull U (1985). Refractive index of water and its dependence on wavelength, temperature, and density. J. Phys. Chem. Ref. Data.

[33] Geints Y, Minin OV, Minin IV (2018). Systematic study and comparison of photonic nanojets produced by dielectric microparticles in 2D- and 3D- spatial configurations. J. Opt..

[34] Jung S, Tiwari MK, Doan NV, Poulikakos D (2012). Mechanism of supercooled droplet freezing on surfaces. Nat. Commun..

[35] Vu TV, Dao KV, Pham BD (2018). Numerical simulation of the freezing process of a water drop attached to a cold plate. J. Mech. Sci. Technol..

[36] Nauenberg M (2016). Theory and experiments on the ice–water front propagation in droplets freezing on a sub-zero surface. Eur. J. Phys..

[37] Vu TV, Ho NX (2022). Numerical study of a hollow pileup yielded by deposition of successive hollow droplets. Phys. Fluids.

[38] Graebera G, Schutziusa TM, Eghlidia H, Poulikakos D (2017). Spontaneous self-dislodging of freezing water droplets and the role of wettability. PNAS.

[39] La S, Huang Z, Liu C, Zhang X (2018). Morphology of supercooled droplets freezing on solid surfaces. AIP Adv..

[40] Irajizad P, Nazifi S, Ghasemi H (2019). Icephobic surfaces: Definition and figures of merit. Adv. Colloid Interface. Sci..

[41] Hakimian, A., Nazifi, S. & Ghasemi, H. Chapter 3. Physics of Ice Nucleation and Growth on a Surface. In: Mittal, K. L., Choi, C.-H. (2020). Scrivener Publishing LLC. 10.1002/9781119640523.ch3

[42] Snoeijer JH, Brunet P (2012). Pointy ice-drops: How water freezes into a singular shape. Am. J. Phys..

[43] Karim OA, Haymet ADJ (1988). The ice/water interface: A molecular dynamics simulation study. J. Chem. Phys..

[44] Zhu Z, Zhang X, Zhao Y, Huang X, Yang C (2022). Freezing characteristics of deposited water droplets on hydrophilic and hydrophobic cold surfaces. Int. J. Therm. Sci..

[45] McCraney J, Ludwicki J, Bostwick J, Daniel S, Steen P (2022). Coalescence-induced droplet spreading: Experiments aboard the International Space Station. Phys. Fluids.

[46] Sibley DN, Llombart P, Noya EG, Archer AJ, MacDowell LC (2021). How ice grows from premelting films and water droplets. Nat. Commun..

[47] Seguy L, Protiere S, Huerre A (2023). Role of geometry and adhesion in droplet freezing dynamics. Phys. Rev. Fluids.

[48] Nauenberg M (2014). Theory and experiments on the ice-water front propagation in droplets freezing on a subzero surface. Eur. J. Phys..

[49] Backholm M (2020). Water droplet friction and rolling dynamics on superhydrophobic surfaces. Commun. Mater..

[50] Tang X (2023). Multifunctional droplet-surface interaction effected by bulk properties. Droplet..

[51] Singh DP, Singh JP (2013). Delayed freezing of water droplet on silver nanocolumnar thin film. Appl. Phys. Lett..

[52] Poggi E, Gohy J (2017). Janus particles: From synthesis to application. Colloid Polym. Sci..

[53] Minin IV, Minin OV (2022). Mesotronics: Some new unusual optical effects. Photonics.

[54] Vollmer M, Möllmann K-P (2013). Is there a maximum size of water drops in nature?. Phys. Teach..

[55] Yue L, Minin OV, Wang Z, Monks J, Minin IV (2018). Photonic hook: A new curved light beam. Opt. Lett..

[56] Christodoulides, D. N. Foreword vii–viii. In: Minin, O. V., Minin, I. V. The Photonic Hook. Springer, Cham (2021).

[57] Bronshtein IN, Semendyayev KA, Musiol G, Muehlig H (2004). Handbook of Mathematics.

[58] Park J, Han HS, Kim YC (2015). Direct and accurate measurement of size dependent wetting behaviors for sessile water droplets. Sci. Rep..

[59] Pan D, Wan Q, Galli G (2014). The refractive index and electronic gap of water and ice increase with increasing pressure. Nat. Commun..

[60] Al-Sharafi A, Yilbas BS, Ali H (2018). A water droplet pinning and heat transfer characteristics on an inclined hydrophobic surface. Sci. Rep..

[61] Gu G, Shao L, Song J, Qu J, Zheng K, Shen X, Peng Z, Hu J, Chen X, Chen M, Wu Q (2019). Photonic hooks from Janus microcylinders. Opt. Express.

[62] Minin IV, Liu C-Y, Geints YE, Minin OV (2020). Recent advances in integrated photonic jet-based photonics. Photonics.

[63] Klein A, Yaron T, Preter E, Duadi H, Fridman M (2017). Temporal depth imaging. Optica.

[64] Klein A, Sibony I, Meir S, Duadi H, Sander MY, Fridman M (2020). Temporal imaging with a high filling-factor. APL Photon..

[65] Stan CA, Kalita A, Marte S, Kaldawi TF, Willmott PR, Boute S (2023). Rocket drops: The self-propulsion of supercooled freezing drops. Phys. Rev. Fluids.

[66] Shayunusov D, Eskin D, Balakin BV, Chugunov S, Johansen ST, Akhatov I (2021). Modeling water droplet freezing and collision with a solid surface. Energies.

[67] Wang C, Xu Z, Zhang H, Zheng J, Hao P, He F, Zhang X (2022). A new freezing model of sessile droplets considering ice fraction and ice distribution after recalescence. Phys. Fluids.

[68] UN Water, “World Water Day,” 2021.

[69] Chen X, Wu T, Gong Z, Li Y, Zhang Y, Li B (2020). Subwavelength imaging and detection using adjustable and movable droplet microlenses. Photon. Res..

[70] Frumkin V, Bush JWM, Papatryfonos K (2023). Superradiant droplet emission from parametrically excited cavities. Phys. Rev. Lett..

[71] J. T. Marmolejo, A. Canales, D. Hanstorp, and R. M´endez-Fragoso. Fano Combs in the Directional Mie Scattering of a Water Droplet. Phys. Rev. Lett., 130, 043804 (2023) 10.1103/PhysRevLett.130.043804 36763447

