# Supplementary material for: Time domain self-bending photonic hook beam based on freezing water droplet
Source: Sci Rep. 2023 May 12;13:7732. doi: 10.1038/s41598-023-34946-7 (PMC10182040; doi:10.1038/s41598-023-34946-7)
Supplement: Supplementary file 1 — Supplementary Legends. [file 41598_2023_34946_MOESM1_ESM.docx]

**supplementary video legends**

Supplement Video 1: The dynamics of the time-PH formation for the drop with radius R=2.5um and with the water-ice interface curvature radius R_c_ =1.5R.

Supplement Video 2: The dynamics of the time-PH formation for the drop with radius R_c_ =3R.
